# Rabies in Nonhuman Primates and Potential for Transmission to Humans: A Literature Review and Examination of Selected French National Data

**DOI:** 10.1371/journal.pntd.0002863

**Published:** 2014-05-15

**Authors:** Philippe Gautret, Jesse Blanton, Laurent Dacheux, Florence Ribadeau-Dumas, Philippe Brouqui, Philippe Parola, Douglas H. Esposito, Hervé Bourhy

**Affiliations:** 1 Assistance Publique Hôpitaux de Marseille, CHU Nord, Pôle Infectieux, Institut Hospitalo-Universitaire Méditerranée Infection, Marseille, France; 2 Aix Marseille Université, Unité de Recherche en Maladies Infectieuses et Tropicales Emergentes (URMITE), UM63, CNRS 7278, IRD 198, Inserm 1095, Faculté de Médecine, Marseille, France; 3 Poxvirus and Rabies Branch, Division of High-Consequence Pathogens and Pathology, National Center for Emerging and Zoonotic Infectious Disease, Centers for Disease Control and Prevention, Atlanta, Georgia, United States of America; 4 Institut Pasteur, Unité Dynamique des lyssavirus et adaptation à l'hôte, National Reference Centre for Rabies, WHO Collaborating Centre for Reference and Research on Rabies, Paris, France; 5 Division of Global Migration and Quarantine, National Center for Emerging and Zoonotic Infectious Diseases, Centers for Disease Control and Prevention, Atlanta, Georgia, United States of America; University of Oklahoma Health Sciences Center, United States of America

## Abstract

**Background:**

The nonhuman primate (NHP)-related injuries in rabies-enzootic countries is a public health problem of increasing importance. The aims of this work are to collect data concerning rabies transmission from NHPs to humans; to collate medical practices regarding rabies postexposure prophylaxis (PEP) in different countries, and to provide an evidence base to support the decision to apply rabies PEP in this context.

**Methodology:**

To retrieve information, we conducted a literature search from 1960 to January 2013. All reports of rabies in NHPs and rabies transmission to humans by infected NHPs were included. Also included were studies of travelers seeking care for rabies PEP in various settings.

Data collected by the French National Reference Centre for Rabies concerning NHPs submitted for rabies diagnosis in France and human rabies exposure to NHPs in travelers returning to France were analyzed for the periods 1999–2012 and 1994–2011, respectively.

**Principal findings:**

A total of 159 reports of rabies in NHPs have been retrieved from various sources in South America, Africa, and Asia, including 13 cases in animals imported to Europe and the US. 134 were laboratory confirmed cases. 25 cases of human rabies following NHP-related injuries were reported, including 20 from Brazil. Among more than 2000 international travelers from various settings, the proportion of injuries related to NHP exposures was about 31%. NHPs rank second, following dogs in most studies and first in studies conducted in travelers returning from Southeast Asia. In France, 15.6% of 1606 travelers seeking PEP for exposure to any animal were injured by monkeys.

**Conclusions/significance:**

Although less frequently reported in published literature than human rabies, confirmed rabies cases in NHPs occur. The occurrence of documented transmission of rabies from NHPs to human suggests that rabies PEP is indicated in patients injured by NHPs in rabies-enzootic countries.

## Author Summary

No international consensus or even a consensus among existing national recommendations about rabies postexposure prophylaxis (PEP) following a nonhuman primate (NHP)-related injury currently exists. Epidemiologic studies and reports collated in this review indicate that the number of rabies case reported in NHPs are rare compared with humans. This finding might be because of a lower contact rate of NHPs with rabid reservoir but also very likely because of underreporting. Nevertheless, documented cases and subsequent transmission to humans have been reported from various sources in South America, Africa, and Asia. Further, international travelers often report NHP-related injuries and NHPs can be close to humans. Little is currently known of the pathobiology of rabies virus shedding in primates, which implies that rabies PEP and administration of rabies immunoglobulin should be considered in patients with a possible exposure.

## Introduction

Among wildlife, nonhuman primates (NHPs) are known to harbor a large diversity of zoonotic pathogens and are among the primary mammals targeted for zoonotic disease surveillance [1]. They are the principal host and sometimes an important intermediate host of many zoonotic RNA viruses. Among these viruses, rabies virus, the agent of a lethal encephalitis, is responsible for around 55,000 human deaths every year [2]. Human rabies is a fatal disease once clinical signs develop. Rabies postexposure prophylaxis (PEP) consists of thorough wound care, in combination with rabies vaccine and administration of rabies immunoglobulin (RIG) if necessary. Despite evidence of rabies virus spillover in NHPs and of transmission of rabies from NHPs to humans, neither the World Health Organization (WHO) nor the United States Centers for Disease Control and Prevention (CDC) provide specific guidelines regarding rabies PEP following NHP-related injuries. Guidance emphasizes the role of most frequent reservoirs and vectors. The recommendation of WHO is to provide vaccine and RIG in severe, type III injuries (transdermal bites or scratches, lick on broken skin or mucous membrane, and contacts with bats) and vaccine only in minor, type II injuries (minor scratches or abrasions without bleeding) following exposure from any wild mammal (including implicitly NHPs) in a previously unvaccinated person [2]). At the international level, PEP recommendations after exposure to various animals may differ across a variety of organizations. This is also the case for recommendations following exposure to NHPs. The human animal interactions are too complicated to list every scenario or most species, given the diversity of mammalian species. Hence, the US CDC recommends that vaccine and RIG be provided, regardless of the type of injury, following exposure from any wild mammal (including implicitly NHPs) for a previously unvaccinated person exposed to rabies, as evaluated based on risk assessment [3]. The US Advisory Committee on Immunization Practices and the National Association of State Public Health Veterinarians deal with risk assessments and particular taxa, on a case-by-case basis. Quebec Province (Canada) guidelines recommend the use of vaccine and RIG following NHP-related injuries [4]. French guidelines recommend following the WHO guidelines [5]. Currently, neither the British nor Scottish guidelines recommend the use of RIG for PEP following NHP-related injuries. The British guidelines state that “rabies-infected primates have been sporadically described in countries where rabies is endemic. Although the risk of transmission of rabies from a primate bite is extremely low, these bites occurring in low- or high-risk countries should receive PEP with vaccine only for a previously unvaccinated subject” [6]. The Scottish guidance document, published in 2010, states that all bites, licks and scratches from NHPs are considered low risk and therefore “5 active vaccinations plus no RIG” is the suggested PEP response for a previously unvaccinated person [7].

Therefore, no international consensus has been reached, even among national recommendations about rabies PEP following a NHP-related injury. Furthermore, none of the guidelines that we reviewed are based on published data about rabies in NHPs and subsequent transmission to humans. To enhance the specificity and scientific basis of future recommendations and guidelines, we gathered information on rabies in NHPs and human rabies cases and exposures following NHP-related injuries. The aims of this work are to 1) collect and analyze data concerning rabies transmission from NHPs to humans, 2) collate medical practices regarding rabies PEP in different countries, and 3) provide an evidence base to support the decision to apply rabies PEP in this context.

## Methods

We searched for all accessible publications and reports containing relevant information on rabies in NHPs and human rabies and rabies exposure and PEP following NHP-related injuries. We also analyzed selected data concerning NHPs submitted for rabies diagnosis in France and rabies PEP following NHP-related injuries sustained by French international travelers.

### Search strategy

To retrieve information, we conducted a literature search from 1960 to January 2013, using the MEDLINE and SCOPUS databases, and cross-referenced the following terms: “rabies,” “nonhuman primates,” and “monkey.” We also used these same search terms to conduct a Google search over the same period. We systematically scanned meeting reports from the Southern and Eastern African Rabies Group (SEARG). We also scanned the reference lists and bibliographies of all material identified from these searches for potentially relevant primary studies that could be included in the review.

### Inclusion criteria

We considered all types of reports in English, French, Spanish, or Portuguese language, with the exception of NHP experimental laboratory studies. All reports of rabies in NHPs and rabies transmission to humans by infected NHPs were included, whether clinically diagnosed or biologically confirmed. Also included were studies of travelers seeking care for rabies PEP in various settings.

### Analysis of data concerning NHPs submitted for rabies diagnosis in France and of French national rabies postexposure prophylaxis data

In France, veterinary and medical doctors collaborate closely to detect cases and organize the medical responses to rabies. On the one hand, dogs and cats responsible for human exposure are kept under veterinary surveillance, when possible. If the animal dies for any reason, laboratory diagnostics are performed to rule out rabies. On the other hand, primary health-care management of patients seeking rabies PEP is delivered through an official national network of Antirabies Medical Centers distributed throughout the country [8]. All data collected by veterinarians and medical doctors are collected and analyzed by the French National Reference Centre for Rabies (NRCR), at Institut Pasteur in Paris.

Data collected by the NRCR concerning NHPs submitted for rabies diagnosis in France and human rabies exposure to NHPs in travelers returning to France were analyzed for the periods 1999–2012 and 1994–2011, respectively.

## Results

### South America (Appendix S1, Tables 1 and 2)

**Table 1:** Human rabies^1^ cases following nonhuman primate-related injuries.

Country of exposure	Year	Animal	number of human cases	References
***America***				
Brazil (States of Ceará and Piauis)^1^	1980–2008	Marmoset	20	9,10
***Asia***				
India (Australian traveler)^2^	1988	Monkey^5^	1	24
India^3^	1998	Monkey^5^	1	20
India^3^	1999	Monkey^5^	1	23
India (German traveler)^4^	2004	Monkey^5^^(^NB/had also contacts with dogs)	1	25
Sri Lanka^3^	1975	Monkey^5^	1	22

1 confirmed by molecular analysis.

2 confirmed by histological observation of Negri bodies in the brain.

3 rabies diagnosis was assessed on clinical criteria only.

4 confirmed by fluorescent antibody testing of brain samples, molecular analysis and mouse inoculation with brain material.

5 species not stated.

**Table 2:** Confirmed rabies in imported nonhuman primates.

Country of importation	Year	Animal (number of cases)	Country of origin	Reference
US^1^	1929	Monkey^6^	Not stated	12
US^1^	1936	Monkey^6^	Not stated	12
US^1^^,^ 2	1947	Ringtail (*Cebus* spp.)	Colombia	12
US^1^^,^ 2	1955	Cynomolgus (*Macacca fasicularis*)	Philippines	12
US^2^^,^ 3	1961	Squirrel monkey (*Siamiri sciureus*)	Peru	12
US^1^^,^ 2 ^,^ 3	1963	Squirrel monkey (*Siamiri sciureus*	Peru	12
US^1^^,^ 2 ^,^ 3	1963	Squirrel monkey (*Siamiri sciureus*	Peru	12
UK^1^^,^ 2	1965	Rhesus (*Macaca mulatta)*	India	21
US^2^^,^ 3	1972	Capuchin monkey	Not stated	Center for Disease Control, 1972 (internal report)
US^2^^,^ 3	1972	Chimpanzee	Sierra Leone	19
US^2^^,^ 3	1974	Marmoset (*Saguinus nigricollis*)	Peru	Center for Disease Control, 1976 (internal report), 13
France^4^	1989	Common macaque (*Macaca sylvana*)	Morocco	National Reference Center for Rabies- France 1989 (unpublished report)
France^5^	1989	Common macaque (*Macaca sylvana*)	Morocco	National Reference Center for Rabies- France 1989 (unpublished report)

1 confirmed by histological observation of Negri bodies in the brain.

2 confirmed by mouse inoculation with brain material.

3 confirmed by fluorescent antibody testing of brain samples.

4 This monkey had been vaccinated with a modified live-virus rabies vaccine of avian origin, 13 days before the onset of symptoms. The viral isolate from the rabid monkey had characteristics consistent with an egg-adapted vaccine strain suggesting that the monkey's infection was vaccine-induced. These included a short incubation period in mice (4–5 days), absence of fluorescent rabies antibodies detectable virus in salivary glands and corneas of the mice, only rare inclusions typical of Negri bodies produced on mouse passage, and high titered growth in eggs on first passage.

5 These monkeys had been vaccinated with a modified live-virus rabies vaccine (strain ERA) 43 and 28 days before the onset of the symptoms, suggesting that the monkey's infection was vaccine induced, although sequencing or typing were not done.

6 species not stated.

Rabies in NHPs is well described in Northeast Brazil in Rio Grande do Norte, Ceará, Piaui and Pernabucco States, where rabies cases were documented in marmosets (Appendix S1). These monkeys are highly adaptable to different habitats and can be found on plantations and in urban parks. They are also commonly captured and kept as pets. A new antigenic variant of rabies virus was identified in marmosets and humans bitten by marmosets, which strongly suggests, in conjunction with surveillance data, that these viruses represent a unique, independent rabies endemic cycle [9]. According to the Brazilian Ministry of Health, over the last three decades 20 human rabies cases were reported following marmoset-related injuries in Ceará and Piaui States [9], [10]. In recent years, antibodies against rabies have also been found in capuchin monkeys in southeastern Brazil in the state of São Paulo [10], and 2 rabies cases were recorded from the same state in monkeys for which the species was not documented, according to the Pan American Health Organization (PAHO) epidemiological information system. Finally, 4 rabies cases were reported in monkeys (species not available) from Mato Grosso in 2010–2011 according to PAHO. In Peru, rabies cases were suspected in humans following pet monkey bites (species not available) from 1999 to 2006 in the region of Lima, although monkeys tested positive by serology, further laboratory investigations led to the conclusion of false positive [11]. Three rabies cases were documented in squirrel monkeys imported from Peru to the United States in the early 1960s [12], as well as one in a marmoset where infection was very likely vaccine-induced [13].

Rabies cases were reported sporadically in monkeys in Argentina, Bolivia, Colombia, Cuba, Ecuador, and Paraguay, according to PAHO. One case was documented in a ringtail monkey imported from Colombia to the United States in 1947 [12].

It must be pointed-out that no information is provided about the diagnostic criteria that were used for cases reported by PAHO.

### Africa (Appendix S1, Table 2)

Data published in the medical literature about rabies in African NHPs are scant [14]–[18]. Meeting reports of the SEARG (web site: http://searg.info/doku.php?id=start) provide some evidence of rabies in NHPs in a number of African countries, including Ethiopia, Ghana, Kenya, Madagascar, Malawi, Mozambique, Namibia, Sudan, Uganda, and Zambia (Appendix S1). The species of primate in these reports is rarely documented. However, cases were reported in baboons, a gorilla, a bush baby, a vervet monkey, and lemurs. One case was reported in a chimpanzee imported from Sierra Leone to the United States in 1972 [19]. In France, 61 NHPs suspected of rabies were submitted for diagnosis to the NRCR, at Institut Pasteur, from 1999 to December 2012. Nine (14.5%) of these animals were sent directly from rabies-enzootic African countries to the NRCR for diagnosis or illegally imported from Africa to France and submitted for rabies diagnosis by the French veterinary services. None were found positive. The last two positive cases were in two common macaques (*Macaca sylvana*) vaccinated with a modified live-virus rabies vaccine (strain ERA) 43 and 28 days before the onset of the symptoms, suggesting that the monkey's infection was vaccine-induced. More than 50 people were exposed to these monkeys and received rabies PEP. Despite intensive searches, we were unable to find a documented human rabies case following exposure from an African NHP.

### Asia and the Middle East (Appendix S1, Tables 1 and 2)

Few published results about rabies in NHPs in Asia are available. Unfortunately, country reports about animal rabies in Asia that can be found in reports of symposium on rabies control in Asia co-organized by the Mérieux Foundation and the WHO do not address NHPs specifically. Rabies cases were reported in monkeys, langurs, and baboons in India [20], including one case in a macaque imported to London in 1965 for laboratory experiments [21]. One case was reported in a macaque imported from the Philippines to the United States in 1955 [12]. Rare human rabies cases following monkey bites have been reported in local populations in India and Sri Lanka, based on clinical diagnosis [20], [22], [23] and in two travelers returning from India to Australia and Germany, based on histopathology in the first case and direct immunofluorescence and virus isolation in the second case [24], [25]. One case was documented in a pet monkey in Jordan [26]. In France, only one NHP imported from Indonesia was submitted for rabies diagnosis to the NRCR from 1999 to December 2012, and it was found negative.

### NHP-related injuries requiring rabies PEP in travelers (Table 3)

**Table 3:** Proportion of injuries caused by nonhuman primates among international travelers injured by potentially rabid animals.

Study period	Place of exposure	Population	Design of the study	Total number of injured travelers (all animal species)	Proportion of nonhuman primate related injuries in travelers	References
Feb 1987–Jan 1989	Nepal	Non-Indian expatriates and tourists presenting at the Katmandu CIWEC Clinic (main clinic for foreigners in Nepal)	Observational survey	51	19.2%	27
Jan 1996–Dec 1998	Nepal	Non-Indian tourist presenting at the Katmandu CIWEC Clinic (main clinic for foreigners in Nepal).	Observational survey	56	43.0%	28
Jul 1998–Mar 2005	Nepal	Expatriates and travelers presenting at the Katmandu CIWEC Clinic (main clinic for foreigners in Nepal)	Retrospective survey	544	27.9%	29
Aug–Dec 2004	Mainly Asia	Israeli travelers (traveling one month and over)	Cohort survey (815 individuals)	13	30.8%	30
June 1998–May 2005	Mainly Asia, Latin America and Africa	Travelers seen after travel at GeoSentinel sites	Multicentric international retrospective survey	321	21.2%	31
May 1997–May 2005	Mainly Africa and South-East Asia	Injured travelers returning to Marseille (France), Melbourne (Australia) and Auckland (New-Zealand)	Retrospective survey	261	17.3%	32
Oct 1998–Feb 2006	Mainly South-East Asia	Injured travelers returning to Auckland and Hamilton (New-Zealand)	Retrospective survey	54	18.5%	33
Jan 1994–Dec 2007	Mainly North Africa and Asia	Injured travelers returning to Marseille (France)	Retrospective study	424	19.6%	34
Nov 2008–Mar 2010	Bali, Indonesia	Injured travelers returning to Marseille (France), Melbourne (Australia), Singapore and Auckland (New-Zealand)	Retrospective survey	45	68.9%	35
Jan 2000–Jul 2009	Mainly Asia and Turkey	Injured travelers returning to Liverpool (United Kingdom)	Retrospective survey	139	16.5%	36
Apr 2009–Jul 2010	Mainly Indonesia and Thailand	Injured travelers returning to 3 clinics in Queensland and 1 in Perth (Australia)	Prospective study	65	44.6%	37
Jun 2010–Feb 2011	Mainly Thailand and other South-east Asian countries	International travelers leaving Bangkok (Thailand)	Cross sectional survey	36 with animal species documented (out of 219)	38.9%	38
Sep–Dec 2011	Afghanistan	US military	Retrospective survey	126	7.9%	39
Jan 2008–April 2012	Mainly Indonesia, Thailand, India and China	Potential rabies exposure incidents reported to Public Health Units in the south Brisbane region of Queensland, (Australia)	Prospective study	136	55.8%	40

**Figure 1:**
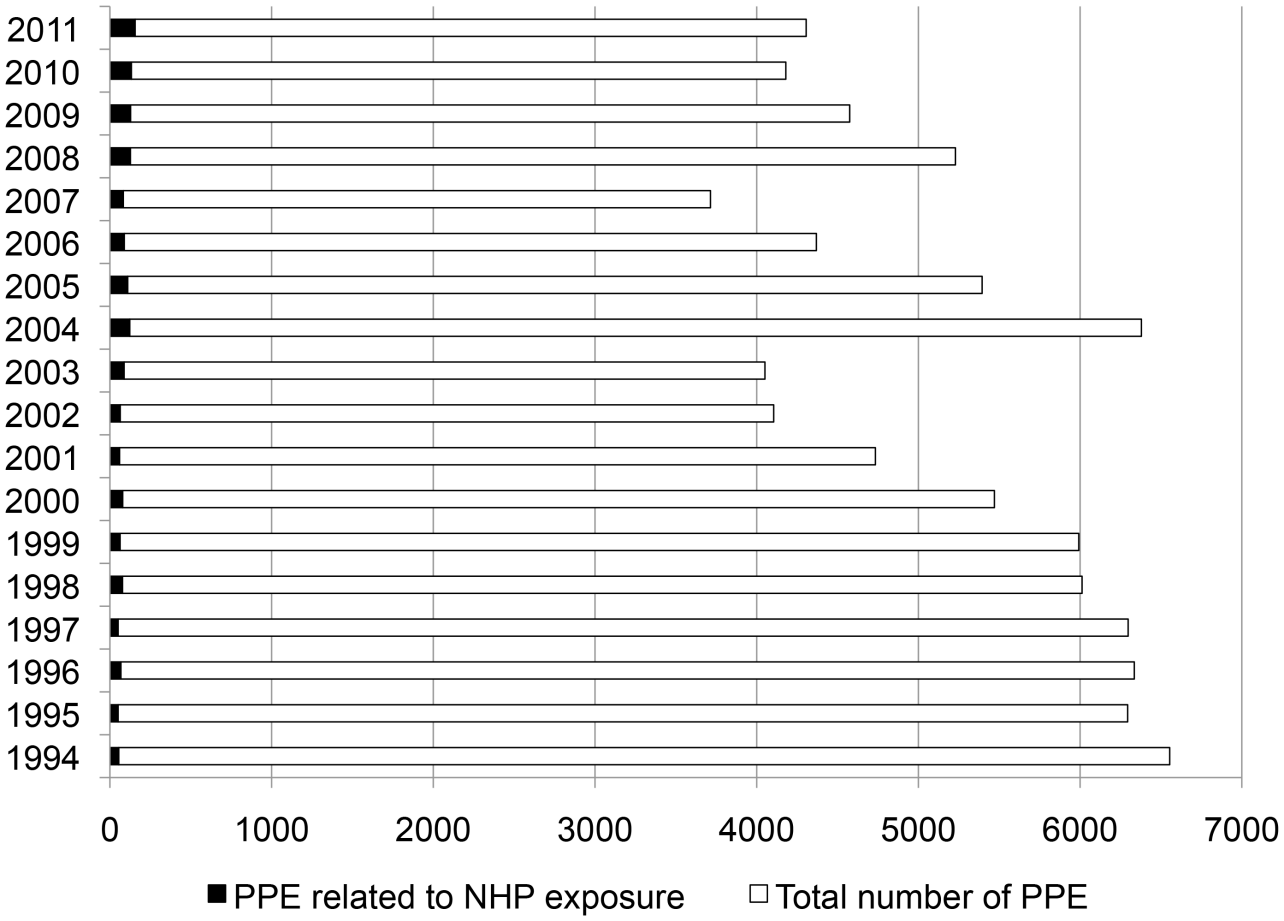
Rabies postexposure prophylaxis (PEP) in France, 1994–2011. NHP = nonhuman primates.

A number of studies were conducted in travelers seeking care for rabies PEP in various settings [27]–[40]. Data are available from more than 2000 people, and the proportion of injuries related to NHP exposures is about 31%, with the smallest proportion observed in US military personnel stationed in Afghanistan (8%) and the largest reported from travelers returning from Bali, Indonesia, at various GeoSentinel clinics (69%). Overall, dogs are usually the most frequently reported species responsible for injuries requiring rabies PEP in travelers. However, NHPs rank second in most studies and first in studies conducted in travelers returning from Southeast Asia (34,35,37,40). In France, data are available from 1606 travelers exposed to NHPs from 1994 to 2011, representing 1.7% of the total number of people and 15.6% of travelers seeking PEP in France for exposure to any animal, during the same period. The number of travelers exposed to NHPs and receiving PEP in France has increased since 2002, especially in 2004 and 2005 (Figure 1) because of a strong demand for antirabies prophylaxis following a well-publicized rabies case in a dog imported to France in 2004 [8]. This proportion increased to 3.1% by 2008–2011 (Figure 1), further indicating that the NHP related injuries in rabies-enzootic countries is a public health problem of increasing importance. The largest proportion of travelers exposed to NHPs and receiving PEP in France during the period 1994–2012 had returned from Asia and the Middle East (53.3%), followed by Africa (36.9%) and the Americas (5%). In Asia and the Middle East, the most frequent country of exposure was Thailand (22.4% of the treated patients).

## Discussion

We retrieved a total of 134 confirmed cases of rabies in NHPs which have been reported from various sources in South America, Africa, and Asia, including 13 cases in animals imported to Europe and the US. We retrieved 25 cases of rabies transmission to humans following NHP-related injuries, 20 of which occurred in Brazil. Rabies cases in NHP from Brazil were confirmed by genetic analysis [9]. Additionally 4 capuchin monkeys were found with positive serology in southeastern Brazil [10]. By contrast, 21 NHPs from other regions in Latin America were reported rabid by the PAHO with no information about the methods used for the assessment of rabies. It is therefore possible that these so-called “cases” were actually healthy animals with a positive-serology. Such so-called “cases” reported in Peru, finally turned out not to be rabies [11]. We cannot exclude that rabies cases reported in NHPs from São Paulo and Mato Grosso in Brazil and from other countries in South America by the PAHO could be actually healthy animals with positive serology. There are issues with the PAHO data that may contain inaccuracies and should not be considered the gold standard. Imported cases from Peru and Colombia, however were confirmed by fluorescent rabies antibody examination of brain tissue, demonstration of negri bodies on microscopic examination or rabies induced in mice inoculated with brain tissue [12], [13]. Cases reported in wild NHPs in various countries in Africa by the SEARG (Appendix S1) and other authors [14]–[18], in India [20] and Jordan [26], as well as in the imported cases from Sierra Leone [19] India [21] and the Philippines [12] were all confirmed by brain tissue histology, fluorescent antibody testing of brain tissue and mouse inoculation.

The reports collated in this study support the view that confirmed rabies cases in NHPs are rarely reported compared with human rabies cases. In light of numerous biological reports establishing the susceptibility of NHPs to rabies, we might have expected the number of NHPs with rabies to have been greater than observed. Several explanations for this finding are possible.

First, with the possible exception of the cluster of marmosets in Ceará State, Brazil (9), NHPs are not known to be a reservoir for maintaining a rabies virus variant in the wild. Second, given that dogs are a domesticated species, sharing a closer bond and degree of interaction with humans than do NHPs, the difference in the contact rates with dogs may account, in part, for the difference in reported rates of rabies between humans and NHPs. However, NHPs are frequently kept as pets and can be close to humans in some regions. Finally, underreporting of rabies in NHPs is likely to be significant. The passive nature of rabies surveillance likely accounts for underreporting of rabid NHPs. Rules pertaining to the submission of animal specimens for rabies diagnosis and reporting to national authorities are sometimes weak and may only cover the few species considered to be economically important or those most important in terms of public health. Last, rabies cases in NHPs are not notifiable in many countries and as such are not recorded in official statistics.

Underreporting of rabies in NHPs is a major impediment to understanding the epidemiology of this disease and may hinder the development of control strategies. We show that a review of published reports can be an important way to overcome the problem of underreporting and can contribute to the advancement of the understanding of the importance of rabies in NHPs as a potential hazard to humans. Moreover, valuable information exists in internal reports, which is not easily available since it is not indexed in MEDLINE and SCOPUS databases.

More complete and precise information pertaining to rabies in NHPs is needed. This information could be obtained through field surveys. We believe a greater effort should be directed toward coordinating and frequently reviewing the need for rabies PEP after exposure to animal species such as NHPs that are not primary reservoirs of rabies. Information obtained in this way should be regularly collected, updated, and made available to the medical community. To this end, efforts towards greater openness and accessibility of information regarding the incidence of rabies in NHPs and its geographic distribution would provide a much-needed basis for improving and sustaining the public health debate around the risk evaluation of rabies after human exposure to these species.

To address the possibility of reintroduction of rabies through NHPs, countries that are designated as rabies-free should strongly consider permitting their entry only under license. Live animal importations to such countries would benefit from quarantine guideline under conditions approved by governmental veterinary services.

We show that, although rarely reported, documented cases of rabies infections in NHPs and subsequent transmission to humans do occur. Little is currently known about the pathobiology of rabies virus shedding in primates. The occurrence of documented transmission of rabies from NHPs to human suggests that rabies PEP is indicated in patients injured by NHPs in rabies-enzootic countries. We were unable to find any report suggesting failure or death in previously unvaccinated persons who received vaccine without RIG after exposure to NHP, however, rabies status of NHP was not documented in these reports. From a clinical perspective, distinct recommendations are found depending on national guidelines. United Kingdom guideline state that the risk of rabies following NHP-related injury is extremely low and that rabies PEP with vaccine only should be applied in previously unvaccinated people [6], [7]. A contrario, WHO, the US CDC, Canadian and French guideline state that the catastrophic nature of the disease with a nearly 100% mortality rate is what will drive treatment, not the low probability of the disease and that rabies vaccine and RIG should be applied in previously unvaccinated people [2]–[5]. As long as wild life studies addressing the role NHPs play in the disease transmission to humans are not available from various area where human exposure occur and as recommended by WHO, we consider that a precautionary principle should be applied and that RIG should be administered, as with any other animal exposures, despite the large number of doses that would be necessary, even in the setting of a RIG shortage.

Based on our review of published reports, a large number of international travelers sustain NHP-related injuries during their trips. Information about the risks posed by exposure to NHPs in enzootic countries, especially in India and Southeast Asia, should be disseminated to the traveling public to help minimize these injuries and the subsequent need for rabies PEP. Travelers should be encouraged to seek a pretravel medical consultation from their health-care provider 4–6 weeks before travel to discuss if rabies pre-exposure vaccination may be recommended in situations where travel activities may involve a higher potential for contact with animals such as NHPs. Travelers should also be encouraged to seek immediate medical care if injured by an NHP species.

## Supporting Information

Appendix S1 **Rabies in nonhuman primates from the Americas, Africa, Asia and the Middle East.**
(DOCX)
