# Correction: Rabies in Nonhuman Primates and Potential for Transmission to Humans: A Literature Review and Examination of Selected French National Data

**DOI:** 10.1371/journal.pntd.0003799

**Published:** 2015-05-13

**Authors:** Philippe Gautret, Jesse Blanton, Laurent Dacheux, Florence Ribadeau-Dumas, Philippe Brouqui, Philippe Parola, Douglas H. Esposito, Hervé Bourhy

There are several errors throughout the text of this article. The country, India, appears several times instead of the correct country, Thailand. In Table 1, the second county under the heading “Asia” should be Thailand. In the Results section under the subsection “Asia and the Middle East (Appendix S1, Tables 1 and 2)”, India in sentence three should be changed to Thailand. In the fifth sentence of the same subsection, the first mention of India, “…reported in local populations in India…”, should be changed to Thailand. In the last sentence of the first paragraph of the Discussion section, the first mention of India, “…in India [20]…”, should be changed to Thailand.
