# Supplementary material for: Rabies in Nonhuman Primates and Potential for Transmission to Humans: A Literature Review and Examination of Selected French National Data
Source: PLoS Negl Trop Dis. 2014 May 15;8(5):e2863. doi: 10.1371/journal.pntd.0002863 (PMC4022521; doi:10.1371/journal.pntd.0002863)
Supplement: Appendix S1 — Rabies in nonhuman primates from the Americas, Africa, Asia and the Middle East. (DOCX) [file pntd.0002863.s001.docx]

**Appendix S1. Confirmed and suspect rabies^1^ in nonhuman primates from the Americas, Africa, Asia and the Middle East.**

| **Country** | **Years** | **Animal (number of cases)** | **Source** |
| --- | --- | --- | --- |
| ***America*** |  |  |  |
| Argentina  Bolivia | 2002  1999-2008 | Monkey^2^ (1)  Monkey^2^ (5) | PAHO database^3^  PAHO database |
| Brazil (States of Rio Grande do Norte, Ceará, Piaui and Pernabucco ) | 1990-2006 | Marmoset^3^ (28) | Batista-Morais, 2000 ; Favoretto, 2001 ; Favoretto, 2006 Núcleo de Controle das Endemias Transmissíveis por vetores, 2004 ; de Freitas Aguiar, 2011 |
| Brazil (State of Ceará) | 2007-2011 | Monkey^2^ (8) | PAHO database |
| Brazil (State of São Paulo) | 2003-2006 | Capuchin monkeys^4^ (4) | Machado, 2012 |
| Brazil (State of São Paulo) | 2007 | Monkey^2^ (2) | PAHO database |
| Brazil (State of Mato Grosso) | 2010-2011 | Monkey^2^ (4) | PAHO database |
| Colombia | 2000 | Monkey^2^ (1) | PAHO database |
| Cuba | 2007 | Monkey^2^ (1) | PAHO database |
| Dominican Republic | 2006-2007 | Monkey^2^ (3) | PAHO database |
| Ecuador | 1999 | Monkey^2^ (1) | PAHO database |
| Paraguay | 1999-2009 | Monkey^2^ (3) | PAHO database |
| ***Africa*** |  |  |  |
| Ethiopia | 1964-1975  1996-2000 | Monkey^2^ (3)  Monkey^2^ (2) | Fekadu, 1982  Ahmed, 2001 |
| Ghana | 1970-1982 | Monkey^2^ (2) | Addy, 1985 |
| Kenya | 1986  1995 | Monkey^2^ (1)  Baboon^2^ (1)  Gorilla^2^ (1) | Chong, 1993; Karugah, 1997 |
| Madagascar | 1994 | Lemur^2^ (4) | Tsiresy , 1995 |
| Malawi | 1994  2000 | Monkey^2^ (1)  Monkey^2^ (1) | Chimera, 2001 |
| Mozambique | 1987-1991  2005 | Vervet monkey (2)  Monkey^2^ (1) | Dias, 1992; Pinto, 2001; Rodrigues, 2006 |
| Namibia | 1994  1990-2009 | Baboon^2^ (2)  Monkey^2^ (2) | Hübschle, 1995  Magwedere, 2012 |
| Sudan | 1992-2002 | Monkey^2^ (7) | Ali, 2001; Röttcher, 1978 |
| Uganda | 1994  1996  1997  1999 | Monkey^2^ (1)  Monkey^2^ (3)  Monkey^2^ (3)  Monkey^2^ (2) | Rutebarika, 1997; Rutebarika, 2001 |
| Zambia | 1928-1976  1994 | Bush baby^5^(1)  Vervet monkey^6^ (1) Baboon^7^ (1 )  Baboon^2^ (1) | Röttcher, 1978  Munang’andu, 1995 |
| ***Asia and the Middle East*** |  |  |  |
| India | 1993-1999 | Monkeys^2^ (18)  Langurs^2^ (15)  Gibbons^2^ (8) | Panichabhongse, 2001 |
| Jordan | 1982-1995 | Monkey^2,8^ (1) | Al-Qudah, 1997 |

^1^All cases reported in this table were laboratory confirmed at least by histological observation of Negri bodies in the brain, fluorescent antibody testing of brain samples, molecular analysis and or mouse inoculation with brain material. No information however is provided about the diagnostic criteria that were used for cases reported by PAHO and must therefore be considered suspect cases. Notably, there are issues with the PAHO data about cases from the States of São Paulo and Mato Grosso in Brazil and that some suspect their accuracy.

^2^species not stated.

^3^Pan American Health Organization. Epidemiological Information System. <http://siepi.panaftosa.org.br/Panel.aspx?Idioma=i> (accessed 31/12/2012).

^3^*Callithrix jacchus*

^4^*Cebus apella nigritus* (these NHPs were found positive by serology only)

^5^*Galago crassicaudatus*

*^6^Cercopithecus aethiops*

***^7^****Papio* spp.

^8^Pet monkey

**References**

Addy PAK. Epidemiology of rabies in Ghana. In: Kuwert E, Merieux C, Koprowski H, Bogel K, editors. Rabies in the Tropics. Heidelberg: Springer-Verlag; 1985. p. 497-515.

Ahmed YE. Rabies in Ethiopia. Proceeding of the SEARG international symposium, 2001 (http://searg.info/fichiers/articles/2001022025L.PDF) accessed 7 Feb 2013.

Ali YA. Rabies in Sudan. Proceeding of the SEARG international symposium, 2001 (<http://searg.info/fichiers/articles/2001050052L.PDF>) accessed 7 Feb 2013.

Al-Qudah KM, Al-Rawashdeh OF, Abdul-Majeed M, Al-Kani FK. An epidemiological investigation of rabies in Jordan. Acta Vet (Beograd). 1997;47(2-3);129-34.

Batista-Morais N, Neilson-Rolim B, Matos-Chaves HH, de Brito-Neto J, Maria-da-Silva L. Rabies in tamarins (Callithrix jacchus) in the state of Ceará, Brazil, a distinct viral variant? Mem Inst Oswaldo Cruz. 2000 Sep-Oct;95(5):609-10.

Chong WK. Rabies in Kenya. Proceeding of the SEARG international symposium, 1993 (<http://searg.info/fichiers/articles/1993050058L.PDF>) accessed 7 Feb 2013.

Centro Nacional de Epidemiologia. Coordenaçao de Controle de Zoonoses e Animais Peçonhentos. Relatorios da Coordenadoria de Zoonoses e Animais Peçonhentos, Brasılia. Quoted by Machado et al, 2012.

Chimera BAR, Chikungwa PP. Rabies in Malawi. Proceeding of the SEARG international symposium, 2001 (http://searg.info/fichiers/articles/2001030033L.PDF) accessed 7 Feb 2013.

de Freitas Aguiar TD, Chaves Costa E, Rolim BN, Romijn PC, de MoraisNB, da Silva Teixeira MF. Risco de transmissão do vírus da raiva oriundo de sagui (Callithrix jacchus), domiciliado e semidomiciliado, para o homem na região metropolitan de Fortaleza, Estado do Ceará. Revi Soc Bras Med Trop. 2011 mai-jun 44(3):356-63.

Dias P. Rabies in Mozambique. Proceeding of the SEARG international symposium, 1992 (http://searg.info/fichiers/articles/1992024025L.PDF) accessed 7 Feb 2013.

Favoretto SR, de Mattos CC, Morais NB, Alves Araújo FA, de Mattos CA. Rabies in marmosets (Callithrix jacchus), Ceará, Brazil. Emerg Infect Dis. 2001 Nov-Dec;7(6):1062-5.

Favoretto SR, de Mattos CC, de Morais NB, Carrieri ML, Rolim BN, Silva LM, Rupprecht CE, Durigon EL, de Mattos CA. Rabies virus maintained by dogs in humans and terrestrial wildlife, Ceará State, Brazil. Emerg Infect Dis. 2006 Dec;12(12):1978-81.

Fekadu M. Rabies in Ethiopia. Am J Epidemiol. 1982 Feb;115(2):266-73.

Hübschle OJB, Uanguta M. Rabies in Namibia. Proceeding of the SEARG international symposium, 1995(http://searg.info/fichiers/articles/1995048050L.PDF) accessed 7 Feb 2013.

Karugah AK. Proceeding of the SEARG international symposium, 1997 (http://searg.info/fichiers/articles/1997025027L.PDF) accessed 7 Feb 2013.

Núcleo de Controle das Endemias Transmissíveis por vetores—NUEND, Coordenadoria de Apoio ao Desenvolvimento da Atenção à Saúde—CODAS, 1998–2003. Boletim da Secretaria da Saúde do Estado do Ceará, Brasil, 2004. Quoted by Favoretto et al, 2006.

Machado GP, Antunes JM, Uieda W, Biondo AW, Cruvinel TM, Kataoka AP, Martorelli LF, de Jong D, Amaral JM, Hoppe EG, Guerra Neto G, Megid J. Exposure to rabies virus in a population of free-ranging capuchin monkeys (Cebus apella nigritus) in a fragmented, environmentally protected area in southeastern Brazil. Primates. 2012 Jul;53(3):227-31.

Magwedere K, Hemberger MY, Hoffman LC, Dziva F. Zoonoses: a potential obstacle to the growing wildlife industry of Namibia. Infect Ecol Epidemiol. 2012;2; 18365.

Munang’andu HM. Rabies in Zambia. Proceeding of the SEARG international symposium, 1995 (<http://searg.info/fichiers/articles/1995036040L.PDF>) accessed 7 Feb 2013.

Panichabhongse P. The epidemiology of rabies in Thailand. A thesis presented in partial fulfillment of the requirement for the degree of Master of Veterinary Studies. 2001.

Pinto ME. Rabies in Mozambique. Proceeding of the SEARG international symposium, 2001 (http://searg.info/fichiers/articles/2001034037L.PDF) accessed 7 Feb 2013.

Rodrigues F. Rabies in Mozambique. Proceeding of the SEARG international symposium, 2006 (http://searg.info/fichiers/articles/2006038045L.PDF) accessed 7 Feb 2013.

Röttcher D, Sawchuk AM. Wildlife rabies in Zambia. J Wildl Dis. 1978 Oct;14(4):513-7

Rutebarika CS. Rabies in Uganda. Proceeding of the SEARG international symposium, 1997 (http://searg.info/fichiers/articles/1997020024L.PDF) accessed 7 Feb 2013.

Rutebarika CS. Rabies in Uganda. Proceeding of the SEARG international symposium, 2001 (http://searg.info/fichiers/articles/2001055059L.PDF) accessed 7 Feb 2013.

Tsiresy R. Rabies in Madagascar. Proceeding of the SEARG international symposium, 1995 (http://searg.info/fichiers/articles/1995045047L.PDF) accessed 7 Feb 2013.
